# Vitamin D Content of Australian Native Food Plants and Australian-Grown Edible Seaweed

**DOI:** 10.3390/nu10070876

**Published:** 2018-07-06

**Authors:** Laura J. Hughes, Lucinda J. Black, Jill L. Sherriff, Eleanor Dunlop, Norbert Strobel, Robyn M. Lucas, Janet F. Bornman

**Affiliations:** 1 School of Public Health, Curtin University, Bentley WA 6102, Australia; laura.j.hughes@postgrad.curtin.edu.au (L.J.H.); lucinda.black@curtin.edu.au (L.J.B.); j.sherriff@curtin.edu.au (J.L.S.); eleanor.dunlop@postgrad.curtin.edu.au (E.D.); 2 National Measurement Institute, 1/153 Bertie Street, Port Melbourne VIC 3207, Australia; norbert.strobel@measurement.gov.au; 3 National Centre for Epidemiology and Population Health, Research School of Population Health, The Australian National University, Canberra ACT 2600, Australia; robyn.lucas@anu.edu.au; 4 Centre for Ophthalmology and Visual Science, University of Western Australia, Perth WA 6009, Australia; 5 School of Veterinary and Life Sciences, Murdoch University, Murdoch WA 6150, Australia

**Keywords:** liquid chromatography with triple quadrupole mass spectrometry (LC-QQQ), liquid chromatography, triple quadrupole, vitamin D, serum 25-hydroxyvitamin D (25(OH)D), plants, algae

## Abstract

Vitamin D has previously been quantified in some plants and algae, particularly in leaves of the Solanaceae family. We measured the vitamin D content of Australian native food plants and Australian-grown edible seaweed. Using liquid chromatography with triple quadrupole mass spectrometry, 13 samples (including leaf, fruit, and seed) were analyzed in duplicate for vitamin D_2_, vitamin D_3_, 25-hydroxyvitamin D_2_, and 25-hydroxyvitamin D_3_. Five samples contained vitamin D_2_: raw wattleseed (*Acacia victoriae*) (0.03 µg/100 g dry weight (DW)); fresh and dried lemon myrtle (*Backhousia citriodora*) leaves (0.03 and 0.24 µg/100 g DW, respectively); and dried leaves and berries of Tasmanian mountain pepper (*Tasmannia lanceolata*) (0.67 and 0.05 µg/100 g DW, respectively). Fresh kombu (*Lessonia corrugata*) contained vitamin D_3_ (0.01 µg/100 g DW). Detected amounts were low; however, it is possible that exposure to ultraviolet radiation may increase the vitamin D content of plants and algae if vitamin D precursors are present.

## 1. Introduction

Approximately a quarter of Australian adults are deficient in vitamin D (serum 25-hydroxyvitamin D (25(OH)D) < 50 nmol/L) [1]. There is seasonal variation in the prevalence of vitamin D deficiency, with 14% of the adult population deficient in summer and 36% in winter [1]. Certain population groups, such as people with dark skin, those wearing covering for religious or cultural reasons, and people living largely indoors, are at greater risk of deficiency due to inadequate sun exposure, particularly in winter months [2]. Although the major source of vitamin D for humans is cutaneous synthesis of vitamin D_3_ following exposure of the skin to solar radiation [3], when sun exposure is inadequate to maintain vitamin D status, dietary sources make a small but useful contribution [4].

In the Australian food supply, fish, meat, eggs, dairy, and fortified margarine are known sources of vitamin D_3_, while mushrooms are mainly a source of vitamin D_2_ and small amounts of vitamins D_3_ and D_4_ [5,6]. Vitamin D_3_ has been found in shiitake mushrooms, a few algae, and several species of Angiosperms (flowering plants) [7] belonging to the plant families of Cucurbitaceae, Fabaceae, Poaceae, and Solanaceae (Table 1). However, although D_3_ is found in several plants, previous results have come mostly from non-edible leaves rather than the edible portions of the different species, such as fruits or seeds. The hydroxylated metabolite of vitamin D_3_, 25(OH)D_3_, is found in animal products [8,9,10] and likely has a greater biological activity than vitamin D_3_ itself [11,12]. There is some evidence that vitamin D_3_ taken orally is more effective than vitamin D_2_ at increasing levels of 25(OH)D [13,14].

Vitamin D regulates and maintains critical levels of calcium and phosphates in the skeleton of vertebrates by promoting absorption of these nutrients from the gastrointestinal tract. Rickets, the softening of bones in children (or osteomalacia in adults) due to vitamin D deficiency, has been increasing globally [15,16]. Vitamin D deficiency has also been linked to a number of other health conditions besides bone health, including reduced muscle function, autoimmune disease, cardiovascular disease, and some cancers [17,18,19].

The anti-rachitic properties of plants were originally discovered by animal feeding studies [20,21,22], although the active compound in these early studies was later identified as vitamin D_2_ produced from fungal contamination, rather than endogenous to the plants. More recently, high-performance liquid chromatography–ultraviolet (HPLC–UV) with mass spectrometry or liquid chromatography–tandem mass spectrometry (LC–MS/MS) have been used to measure vitamin D metabolites directly in the plant matrix [23,24,25].

This paper focuses on some of the Australian native foods in an effort to detect possible new sources of vitamin D in plants and seaweed. The commercial production of native foods across Australia is estimated to have a gross value of more than 15 million Australian dollars [33]. In a recent stocktake of the Australian native plant industry, it was found that production of lemon myrtle and mountain pepper dominated over other native species, with cultivated supplies of most species [33]. A majority of native food production is used as raw material for value-added products [33].

Given the emerging interest in Australian native food plants for local consumption and export [33], we measured vitamin D_2_, vitamin D_3_, 25-hydroxyvitamin D_2_ (25(OH)D_2_), and 25(OH)D_3_ in a selection of Australian native food plants and Australian-grown edible seaweed. Because the metabolisms of calcium and vitamin D are closely linked in animals and there is a potential link between calcium and vitamin D metabolism in plants [34], we selected plants and seaweed with known high calcium (Ca) content. These included *Acacia victoriae* (wattleseed; 434 mg Ca/100 g, seeds [35]), *Tasmannia lanceolata* (Tasmanian mountain pepper; 495 and 148 mg Ca/100 g, dried leaves and berries, respectively [35])*, Backhousia citriodora* (lemon myrtle; 1583 mg Ca/100 g, dried leaves [35]), and the seaweeds, *Lessonia corrugata* (kombu; 706 mg Ca/100 g, dried [36]) and *Undaria pinnatifida* (wakame; 1100–3000 and 150 mg Ca/100 g, dried and fresh, respectively [36,37]).

Relatively little detailed research has been carried out on Australian native foods, although the existing studies have shown many of the traditional foods to have high nutrient content. For example, the edible seed of the wattleseed tree (from the family Leguminosae) has a strong nutty or coffee-like flavor and has been included in sweet dishes or as a coffee substitute [33]. It is also a good source of calcium, magnesium, and zinc [35]. The lemon myrtle tree, family Myrtaceae, produces leaves with an intense lemon/lemongrass flavor. These leaves are used fresh or dried as a culinary herb, as a tea, or for use in cosmetics and food flavoring agents in the form of extracted oil [33]. The lemon myrtle leaves are high in calcium, vitamin E, and antioxidants [35]. Mountain pepper (from the family Winteraceae), a shrub native to Tasmania, is a versatile plant with both the berries and leaves being used as a food source [33]. The fresh and dried berries are used as an alternative to traditional pepper, and fresh and dried leaves are used as a culinary herb [33]. Both the leaves and berries have been used as therapeutic agents and as a preservative [33]. The plant is high in vitamin E, folate, and antioxidants with moderate levels of calcium [35]. Finally, two types of kelp seaweeds were selected: kombu (family Lessoniaceae), a native of Tasmania [38]; and wakame (family Alariaceae), an introduced kelp species [38,39], hand-harvested in the wild, mostly in Tasmania. Both kombu and wakame are high in calcium [36,37].

With this initial study, we highlight the possibility of using the selected plants and algae as a natural and additional source of vitamin D for reducing the incidence of vitamin D deficiency in vulnerable population groups. For the vitamin D analyses of the selected species, we used liquid chromatography with triple quadrupole mass spectrometry (LC-QQQ), which has been validated for the detection of low levels of vitamin D metabolites in biological samples [40], modified to suit the complex matrices of plants and algae.

## 2. Materials and Methods

### 2.1. Sample Acquisition

Samples of Australian native food plants (wattleseed (*Acacia victoriae*), lemon myrtle (*Backhousia citriodora*), and Tasmanian mountain pepper (*Tasmannia lanceolata*)) and Australian-grown edible seaweeds (wakame (*Undaria pinnatifida*) and kombu (*Lessonia corrugata*)) were sourced from commercial growers or wild harvesters. The selected plants were identified as commonly consumed and commercially available in the Australian food supply [33,35].

The samples were shipped directly from the growers and harvesters to the National Measurement Institute of Australia (NMI), Port Melbourne, Victoria, for preparation and analysis. To maintain their integrity, fresh samples were shipped in an insulated box containing cooler bricks. The details of quantity and source of samples, along with any processing by the growers and harvesters, are outlined in the Supplementary Materials (Table S1).

### 2.2. Sample Preparation

Upon arrival at NMI, dried samples were stored at room temperature, and fresh samples were stored at <5 °C. Dried and freeze-dried samples were homogenized. Fresh samples were prepared as follows: the leaves were cut to 1 cm squares, and fresh fruit was blended; the weight was recorded; the samples were frozen overnight at −70 °C and then freeze dried for 48 h to −50 °C and <10 mTorr; the weight was recorded again; and the freeze-dried factor was determined. Each dried and fresh sample yielded one analytical sample. The prepared samples were stored between −16 °C and −20 °C until extraction and analysis.

### 2.3. Sample Analysis

The instrumentation used was similar to that of Jäpelt et al. [25]. Extraction procedures were derived from published methodology [25,41]. An equivalent deuterated internal standard was added for each vitamin D analogue under investigation: 100 µL of a mixed internal standard solution was added to each sample. This contained 100 ng/mL each of vitamin D_3_ [^2^H_3_], vitamin D_2_ [^2^H_3_], 25(OH)D_3_ [^2^H_3_], and 25(OH)D_2_[^2^H_3_] (Iso Sciences/PM Separations).

The samples were homogenized with 1 g ascorbic acid, 10 mL deionized water, 30 mL absolute ethanol, 2 g potassium hydroxide pellets, and 100 µL of 100 ng/mL deuterated internal standard mix and made to 50 mL with deionized water. The headspace was flushed with nitrogen gas, capped, and placed in a shaker for saponification overnight. The samples underwent centrifugation and 10 mL of the ethanol layer was extracted onto diatomaceous earth Solid Phase Extraction (SPE) cartridges (ChemElute Agilent). The organic soluble compounds were washed off with two 30 mL aliquots of petroleum spirits. The washes were collected into 80 mL glass EPA vials and then evaporated to dryness under high purity nitrogen gas. The residue was reconstituted into 400 µL heptane and transferred to a LC vial containing a 400 µL glass insert. The prepared extracts were stored at −20 °C.

The reagent, PTAD (4-phenyl-1,2,4-triazoline-3,5-dione) reacts non-specifically with dienes under a reaction mechanism called the Diels–Alder Reaction. While in theory, excess PTAD is added to derivatize the vitamin D analogues, samples with high diene content may limit the amount of PTAD available for derivatization. Therefore, where samples were determined to have high diene content, extract clean-up via normal phase chromatography fraction collection was performed. The extracts were inspected for cold precipitate: if present, the liquid extract was transferred to a fresh 400 µL glass insert. Of the remaining liquid extract, 200 µL were injected onto a normal phase chromatographic system with a silica column, 1 mL/min 2% isopropyl alcohol in heptane mobile phase, and a photodiode array detector set to 265 nm. Vitamin D and 25(OH)D fractions were collected.

Fractions of vitamin D and 25(OH)D were combined and evaporated under high purity nitrogen gas. The dry material was reconstituted in 200 µL of dry acetonitrile containing 1 mg/mL of 4-phenyl-1,2,4-triazole-3,5-dione (PTAD) and transferred to a fresh LC vial. Two hours were allowed to complete derivatization. The sample was evaporated under high purity nitrogen gas. The dry material was reconstituted in 100 µL of methanol and water (70:30), transferred to a fresh 400 µL glass insert, and placed into an LC vial. A limit of quantitation was conservatively set at 0.05 µg/100g, which is half of the ‘spiked’ recovery level of 0.1 µg/100g.

The recoveries were determined for each sample analyzed. The recoveries at the 0.1 µg/100 g ‘spiked’ recovery level were as follows: vitamin D_3_, 86–104%; vitamin D_2_, 76–105%; 25(OH)D_3_, 85–114%; and 25(OH)D_2_, 90–114%. The recovery for 25-hydroxyvitamin D_3_ in wattleseed (roasted/milled/ground seed) could not be determined due to a matrix interference.

The samples were analyzed for vitamin D_2_, vitamin D_3_, 25(OH)D_2_, and 25(OH)D_3_ using LC-QQQ (Agilent, San Jose, CA, USA). The calibration samples of vitamin D_2_, vitamin D_3_, 25(OH)D_2_, and 25(OH)D_3_ were prepared. The calibration concentrations (in ng/mL) were 0, 2.5, 5, 7.5, 10, 15, 25, 50, 75, and 100. Each calibration sample also contained 10 ng/mL of deuterated internal standard for each vitamer (vitamin D analogue) tested. The calibrations and samples were analyzed using 1290 Infinity Series LC System/6460 Triple Quad liquid chromatography–tandem mass spectrometry (LC–MS/MS; Agilent Technologies Mulgrave, Victoria, Australia) fitted with a Jet Stream ESI source in positive ion mode using a Supelco Ascentis Express C18 10 cm × 2.1 mm, 2.7 µm LC chromatographic column (Sigma-Aldrich, Sydney Australia).

For each vitamer analyzed, each sample was tested in duplicate, and duplicate values were averaged to obtain one mean value for each sample. A third sample, spiked with the same vitamer, was analyzed for each sample tested to provide quality control data. The mean percentage recovery and mean relative percentage difference were calculated for each vitamer. At the time of writing, the expected limit of detection, post validation study, is expected to be 0.05 µg/100 g (N. Strobel, email communication, 10 October 2017).

The mean recovery percentage across all samples for vitamin D_2_, vitamin D_3_, 25(OH)D_2_, and 25(OH)D_3_ was 96%, 98%, 101%, and 94%, respectively. Across all samples, the mean relative percentage difference between duplicate samples for vitamin D_2_, vitamin D_3_, 25(OH)D_2_, and 25(OH)D_3_ was 71%, 15%, 50%, and 56%, respectively.

## 3. Results

Of the 13 samples tested, three contained quantifiable vitamin D metabolites. Vitamin D_2_ was found in dried lemon myrtle leaves (0.24 µg/100 g) and the dried leaves and berries of Tasmanian mountain pepper (0.67 and 0.05 µg/100 g, respectively). In addition, three samples contained detectable vitamin D metabolites at levels below the limit of quantitation. Approximate levels are provided for indicative purposes only. Vitamin D_2_ was detected in raw wattle seed (≈0.03 µg/100 g) and fresh lemon myrtle leaves (≈0.03 µg/100 g). Vitamin D_3_ was detected in fresh kombu (≈0.01 µg/100 g). There were no detectable vitamin D metabolites in the other samples (Table 2).

## 4. Discussion

We detected low levels of vitamin D_2_ in raw wattleseed, dried leaves and fruit of Tasmanian mountain pepper, and fresh and dried lemon myrtle leaves. Although fungal infection was not tested for in our study, the vitamin D_2_ content found in the plants may have been due to fungal contamination [42]. Vitamin D_2_ is considered a marker for fungal contamination in some crops, such as ryegrass (*Lolium perenne* L.) and hops (*Humulus lupulus* L.) [43,44]. Vitamin D_3_ and, in some cases, 25(OH)D_3_ have previously been detected in the leaves of tomato [24,25,26], waxy leaf nightshade [23,25], potato [24], day blooming jasmine [27], zucchini [24], and alfafa [28]; however, we did not detect these metabolites in our samples of native Australian plants and detected only very low levels in seaweed.

Recently, the fruit of the rimu tree (*Dacrydium cupressinum*), a podocarp native to New Zealand, was found to contain substantial amounts of both vitamin D_2_ and D_3_ [29]. Measured by isocratic reversed-phase HPLC, the average vitamin D_2_ and D_3_ contents of rimu berries were reported as 70 µg/100g and 11.5 µg/100g, respectively, although no quality control data were provided. In another study, the precursors of vitamin D_2_ and D_3_ (ergosterol and 7-dehydrocholesterol, respectively) were detected in plant oils [45]. Other studies have found that the vitamin D_3_ and 25-hydroxyvitamin D_3_ content of leaves and cell cultures of certain plants increases after ultraviolet (UV) irradiation [25,27,46]. For example, exposure to UV radiation increased the vitamin D_3_ content of tomato (*Solanum lycopersicum* L.) leaves by almost 60 times to 100 ng/g, compared with 1.7 ng/g in non-UV-exposed leaves [25]. Future investigations into other potential plant sources of vitamin D and the effect of exposure to UV radiation appear warranted by the finding that consumption of plant oils, particularly UV B-irradiated wheat germ oil, increased 25(OH)D plasma concentration in mice [45].

*Sargassum*, an edible macroalgae [38], was first discovered to have anti-rachitic properties when the lipid fractions of the algae were fed to rats with induced rickets [47]. Since then, vitamin D_2_ and vitamin D_3_ have been found in microalgae and macroalgae using high-performance liquid chromatography (HPLC) in much larger quantities than were found in our study [30,31,32]. Vitamin D metabolites were largely undetected in macroalgae in our study, with the exception of vitamin D_3_ in kombu (*Lessonia corrugata*) measured at 0.01 μg/100 g. In other studies, Japanese wireweed (*Sargassum muticum*) was found to contain 90 μg/100 g, while vitamin D_2_ and D_3_ contents in microalgae ranged from not detected to 3900 μg/100 g and 2.2–271.7 μg/100 g, respectively [30,31,32]. Ergosterol and 7-dehydrocholesterol have also been found in microalgae [31]. As with plants, it has been suggested that the significant vitamin D content of algae is dependent on exposure to UV radiation [30,48]. The role of UV radiation has been implicated by the finding that microalgae harvested in summer have a higher vitamin D_2_ and D_3_ content than those harvested in autumn and winter [30]. Although we detected only low levels of vitamin D_3_ in kombu and no vitamin D_2_ or vitamin D_3_ in wakame, the algae tested in our study were harvested in the winter months and were not sundried or exposed to UV radiation after harvest.

Plant and algal matrices present challenges for the quantification of vitamin D_2_, vitamin D_3_, 25(OH)D_2_, and 25(OH)D_3_, due in part to the presence of interfering compounds such as chlorophyll and lipophilic pigments [48]. Therefore, any method used must be highly sensitive and selective [48]. When compared to single mass spectrometry (MS), LC-QQQ has higher sensitivity and selectivity when applied to the detection of pesticides in water and soil samples [49]. To our knowledge, this method has not been used previously to detect vitamin D metabolites in complex plant and algal matrices and is a major strength of our study due to the low detection limits of the instrumentation. The mean recovery from all spiked samples in our study was high, indicating that LC-QQQ is highly accurate in detecting low levels of vitamin D in plant and algal matrices. All samples were measured in duplicate, and where possible, we tested the edible portion in addition to the leaf material. However, we tested only a few species of Australian native food plants and Australian-grown edible seaweed. Although regional and seasonal variation have been shown to influence the vitamin D content of plants [43,48], we analyzed only single samples sourced from single locations and during months of relatively low UV radiation levels.

In conclusion, this study has demonstrated the high sensitivity of LC-QQQ methodology, which will be used in future studies in search of natural dietary sources of vitamin D. Our results show that the selected Australian native plants and algae have very low levels of vitamin D. However, given that the vitamin D precursors, ergosterol and 7-dehydrocholesterol, have previously been found in both plants and algae, testing the effect of exposure to UV radiation on the vitamin D content of Australian native food plants and Australian-grown edible seaweed is warranted. Also, a larger sample size across a greater number of plant species and habitats will increase the likelihood of identifying nutritionally relevant amounts of vitamin D. An important factor in future studies will be taking the seasonal effect of vitamin D into account in natural ecosystems, because this will relate back to the levels of solar UV radiation for potentially increasing vitamin D levels.

## Figures and Tables

**Table 1:** Content of vitamin D_2_, vitamin D_3_, and 25-hydroxyvitamin D_3_ in plants, microalgae, and macroalgae derived from previously published literature.

Common Name (Botanical Name)	Plant part	Vitamin D_2_ (µg/100 g)	Vitamin D_3_ (µg/100 g)	25(OH)D_3_ (µg/100 g)
**Plants**				
Tomato	Leaf	Not tested	78 (DW) ^a^	2 (DW) ^a^
(*Lycopersicon esculentum*)	Leaf	Not tested	110 (FW) ^b^	1.5 (FW) ^b^
	Leaf	Not tested	0.17 (DW) ^c^	n/d ^c^
Waxy leaf nightshade	Leaf	Not tested	0.32 (DW) ^c^	0.08 (DW) ^c^
(*Solanum glaucophyllum*)	Cell culture derived from leaf material	Not tested	220.00 (FW) ^d^	100.00 (FW) ^d^
Potato	Leaf	Not tested	15 (FW) ^b^	n/d ^b^
(*Solanum tuberosum*)				
Bell pepper	Leaf	Not tested	n/d ^c^	n/d ^c^
(*Capsicum annuum*)				
Day blooming jasmine	Leaf	Not tested	10 (DW) ^e^	10 (DW) ^e^
(*Cestrum diurnum*)				
Zucchini	Leaf	Not tested	23 (FW) ^b^	Not tested
(*Cucurbita pepo*)				
Alfalfa/Lucerne	Leaf	4.8 DW) ^f^	0.06 (DW) ^f^	Not tested
(*Medicago sativa*)				
Rimu (*Dacrydium cupressinum*)	Fruit	70 (DW) ^g^	11.5 (DW) ^g^	Not tested
**Algae**				
*Microalgae*				
Phytoplankton	Whole algae	1.9–4.3 (DW) ^h^	2.2–14.7 (DW) ^h^	Not tested
		5.3 (DW) ^i^	80.4 (DW) ^i^	Not tested
		72.4 (DW) ^i^	271.7 (DW) ^i^	Not tested
(*Pavlova lutheri*)	Whole algae	3900 (DW) ^j^	Not tested	Not tested
(*Tetraselmis suecica*)	Whole algae	1400 (DW) ^j^	Not tested	Not tested
Marine centric diatom (*Skeletonema costatum*)	Whole algae	1100 (DW) ^j^	Not tested	Not tested
(*Isochrysis galbana*)	Whole algae	500 (DW) ^j^	Not tested	Not tested
(*Chaetoceros calcitrans*)	Whole algae	n/d ^j^	Not tested	Not tested
*Macroalgae*				
Japanese Wireweed (*Sargassum muticum*)	Not specified	90 (DW) ^j^	Not tested	Not tested

Decimal places are reported as per the original reference. n/d, not detected; DW: dry weight; FW: fresh weight ^a^ [26] ^b^ [24] ^c^ [25] ^d^ [23] ^e^ [27] ^f^ [28] ^g^ [29] ^h^ [30] ^i^ [31] ^j^ [32].

**Table 2:** New data on the content (dry weight) of vitamin D_2_, vitamin D_3_, 25-hydroxyvitamin D_2_, and 25-hydroxyvitamin D_3_ in Australian native food plants and edible seaweed.

Common name (*Botanical name*)	Food Type	Part Tested	Vitamin D_2_ (µg/100 g)	Vitamin D_3_ (µg/100g)	25(OH)D D_2_ (µg/100 g)	25(OH)D D_3_ (µg/100 g)
Wattleseed	Plant	Leaf	<0.05	<0.05	<0.05	<0.05
(*Acacia victoriae*)		Raw seed	0.03 *	<0.05	<0.05	<0.05
		Roasted, milled seed	<0.05	<0.05	<0.05	<0.05
Tasmanian mountain pepper	Plant	Fresh leaf	<0.05	<0.05	<0.05	<0.05
(*Tasmannia lanceolata*)		Dried leaf	0.67	<0.05	<0.05	<0.05
		Fresh berries	<0.05	<0.05	<0.05	<0.05
		Dried berries	0.05	<0.05	<0.05	<0.05
Lemon myrtle	Plant	Fresh leaf	0.03*	<0.05	<0.05	<0.05
(*Backhousia citriodora*)		Dried Leaf	0.24	<0.05	<0.05	<0.05
Wakame	Algae	Fresh upper leaf and central stem	<0.05	<0.05	<0.05	<0.05
(*Undaria pinnatifida*)		Dried upper leaf and central stem	<0.05	<0.05	<0.05	<0.05
Kombu	Algae	Fresh leaf	<0.05	0.01*	<0.05	<0.05
(*Lessonia corrugata*)		Dried leaf	<0.05	<0.05	<0.05	<0.05

* This result is below the limit of quantitation and is provided for indicative purposes only.

## Supplementary Materials

The following are available online at http://www.mdpi.com/2072-6643/10/7/876/s1, Table S1: Description of plant and algae samples included in the current study.
