# Supplementary material for: Vitamin D Content of Australian Native Food Plants and Australian-Grown Edible Seaweed
Source: Nutrients. 2018 Jul 6;10(7):876. doi: 10.3390/nu10070876 (PMC6073725; doi:10.3390/nu10070876)
Supplement: Supplementary file 1 [file nutrients-10-00876-s001.zip › nutrients-313841-SI.pdf]

Supplementary Materials

Supplementary Table S1. Description of plant and algae samples included in the current study.

Common name (*Botanical name*)	Food type	Plant part	Quantity	Location	Processing	Edible/ Toxic
Wattleseed (*Acacia victoriae*)	Plant	Leaf	1 kg	SA	Picked fresh morning of shipping August 2017	Toxic [a]
		Raw seed	200 g		Harvested January 2017	Edible [a]
		Roasted, milled seed	188 g		Processed as needed, seeds roasted in a rotating drum roaster for 20 min at 400 °C, seeds ground in a grain mill	Edible [a]
Tasmanian mountain pepper (*Tasmannia lanceolata*)	Plant	Leaf	1 kg	TAS	Picked fresh morning of shipping August 2017	Edible [a]
		Dried leaf	200 g		Harvested March–May 2017, warm air-dried	Edible [a]
		Fresh berries	1 kg		Frozen to <4 °C	Edible [a]
		Dried berries	200 g		Harvested March–May 2017, warm air-dried	Edible [a]
Lemon myrtle (*Backhousia citriodora*)	Plant	Leaf	1 kg	NSW	Picked fresh morning of shipping August 2017	Edible [a]
		Dried Leaf	200 g		Machine harvested morning of processing, leaf and sticks separated, leaves dried in an air dryer for 12–24 h	Edible [a]
Wakame (*Undaria pinnatifida*)	Algae	Fresh upper leaf and central stem	1 kg	TAS	Chilled to 4 °C after harvest August 2017	Edible [b]
		Dried upper leaf and central stem	100 g		Chilled to 4 °C after harvest, processed <48 h after harvest, blanched 45 s at 70 °C, plunged into ice water, drained, air-dried	Edible [b]
Kombu (*Lessonia corrugata*)	Algae	Fresh leaf	1 kg	TAS	Chilled to 4 °C after harvest August 2017	Edible [b]
		Dried leaf	100 g		Chilled to 4 °C after harvest, processed <48 h after harvest, chopped, air-dried	Edible [b]

SA, South Australia; TAS, Tasmania; and NSW, New South Wales. [a] Hegarty, M.P.; Wills, R.B.H. Food safety of Australian plant bushfoods. Rural Industries, Research Development Corporation: Barton, A.C.T., 2001. [b] Sanderson, J.C.; Di Benedetto, R. Tasmanian seaweeds for the edible market. Department of Sea Fisheries, Marine Laboratory: Taroona, Tasmania, 1988.
